# Selection and validation of genes related to oxidative stress production and clearance in macrophages infected with *Mycobacterium tuberculosis*

**DOI:** 10.3389/fcimb.2023.1324611

**Published:** 2023-12-12

**Authors:** Renchun Su, Jinfeng Yuan, Tianhui Gao, Yuhong Liu, Wei Shu, Yufeng Wang, Yu Pang, Qi Li

**Affiliations:** ^1^ Department of Bacteriology and Immunology, Beijing Chest Hospital, Capital Medical University/Beijing Tuberculosis and Thoracic Tumor Research Institute, Beijing, China; ^2^ Department of Infectious Diseases, Beijing Chaoyang Hospital, Capital Medical University, Beijing, China; ^3^ Clinical Center on Tuberculosis Control, Beijing Chest Hospital, Capital Medical University/Beijing Tuberculosis and Thoracic Tumor Research Institute, Beijing, China

**Keywords:** *Mycobacterium tuberculosis*, oxidative stress, macrophages, transcriptome, genes

## Abstract

**Background:**

In the fight against tuberculosis, besides chemotherapy, the regulation of oxidative stress (OS) has also aroused people’s interest in host-oriented therapy. However, there is limited research on the genes involved in reactive oxygen species (ROS) production and clearance in macrophages infected with *Mycobacterium tuberculosis* (MTB). This study analyzes and explores this to provide a basis for exploring new targets for antituberculosis treatments.

**Methods:**

We established a macrophage model infected with MTB, counted intracellular bacteria, and determined the ROS produced using flow cytometry. We conducted ribonucleic acid sequencing, screened differentially expressed genes through transcriptomic methods, and validated the expression of them through reverse transcription-quantitative polymerase chain reaction.

**Results:**

The ROS of macrophages increased with intracellular bacteria at 4 h after infection with MTB and reached its peak at 48 h, surpassing the uninfected macrophages (*p* < 0.05). A total of 1,613 differentially expressed genes were identified after infection with MTB, of which 458 were associated with ROS, with over 50% involved in the response of organelles and biological processes to stimuli. We analyzed and identified six genes. After macrophage infection with MTB, the expression of *CAMK2B* increased, whereas the expression of *CYBB* decreased (*p* < 0.05). The expression of *GPX3* and *SOD2* increased, whereas the expression of *CAT* decreased (*p* < 0.05).

**Conclusion:**

The ROS-related differentially expressed genes between MTB infected and uninfected macrophages may be related to some organelles and involved in various biological processes, molecular functions, and signaling pathways. Among them, *CAMK2B, GPX3*, and *SOD2* may be related to ROS.

## 1 Introduction

Tuberculosis remains the most serious threat to global public health and the incidence has increased again over the past 2 years. A projected 1.6 million persons died from tuberculosis in 2021, up from a projected 10.1 million cases in 2020 (Organization, 2022). One of the leading causes of death from bacterial infectious diseases is tuberculosis.

Oxidative stress (OS), also known as biological oxidation, is caused by an imbalance between oxidation and antioxidation (Sies, 1985). The balance of reactive oxygen species (ROS) and antioxidants may be intimately associated with the onset and progression of illnesses, particularly those that impair the respiratory system (Valko et al., 2007; Zuo and Wijegunawardana, 2021). Macrophages are essential for preserving tissue homeostasis and reacting to pathogenic stimuli. According to research, the infection of macrophages with *Mycobacterium tuberculosis* (MTB) leads to the production of ROS, which may have antituberculosis effects (Shastri et al., 2018). Therefore, research on the OS caused by MTB is crucial for the treatment of tuberculosis. Additionally, there might be additional possibilities to lessen oxidative damage and support antituberculosis treatment due to the advancement of antioxidant drug research (Amaral et al., 2016).

Transcriptomics is a powerful technique for the investigation of disease processes. OS is caused by excessive ROS production. It has also been shown that MTB infection causes ROS (Borbora et al., 2023). Little is known, nevertheless, about the molecular process by which MTB produces ROS.

This study detected ROS levels and used ribonucleic acid sequencing (RNA-seq) to identify differentially expressed genes in macrophages infected with MTB. An analysis of the ROS-related genes in macrophages infected with MTB was then performed, opening the door to further investigations into the causes and mechanisms of OS in these cells.

## 2 Materials and methods

### 2.1 Cultures of bacteria

The H37Rv MTB (ATCC, Manassas, VA, USA) strains were grown in Middlebrook 7H9 broth (BD, Franklin Lakes, NJ, USA) supplemented with 10% oleic acid–albumin–dextrose–catalase (OADC) and 0.05% Tween-80 (Sigma, USA). After 15–20 days of culture, the OD_600_ absorbance of the bacterial solution reached 0.6–1.0 [about 1–3 × 10^8^ colony-forming units (CFU)/ml]. Only H37Rv MTB was used in this experiment, no clinical or drug-resistant strains were used.

### 2.2 Cultures of cells

The human monocytic leukemia cell line (THP-1) came from the American Type Culture Collection (ATCC) and was cultured in Roswell Park Memorial Institute (RPMI) medium (Gibco, Carlsbad, CA, USA) supplemented with 10% fetal calf serum (Gibco) at 37°C in 5% CO_2_. For the experiments, the cells were seeded on a 12-well plate or a 6-well plate. Each well contained 10^6^ and 2 × 10^6^ cells, respectively. The cells were cultured for 36 h in the presence of a phorbol ester (PMA; Multi Sciences, Hangzhou, China; 100 nM) to induce differentiation into macrophages (Lyu et al., 2022; Shah et al., 2022). The cells were washed and incubated for an additional 24 h in a fresh medium without PMA until they were used in experiments. Before infection, approximately 70%–80% of the cells had already adhered and had some expanded pseudopodia, which were then visualized under an optical microscope.

### 2.3 Macrophage infection and quantification of mycobacteria

The cells were seeded on a 12-well plate or a 6-well plate. The MTB was grown to the logarithmic growth phase at OD_600 =_ 0.6. The MTB was collected and washed twice in 1 × phosphate-buffered saline (PBS) buffer. Then, MTB was used to infect macrophages with a multiplicity of infection (MOI) equal to 10: 1 for 3 h at 37°C. At the end of the infection, the plates were refreshed with new cell culture medium and then cultured further.

When the measurement time arrived, culture medium from the cell culture plate was discarded, and a 0.05% sodium dodecyl sulfate (SDS) solution added (AMRESCO, USA). The bacterial culture and cell lysate was continuously diluted and cultured on Middlebrook 7H10 agar (BD) supplemented with 10% OADC. After 3 weeks of incubation at 37°C, the number of intracellular MTB was calculated. 7H10 supplemented with 10% OADC medium is a classic medium for the isolation and cultivation of MTB and has been used in numerous studies (Bemer-Melchior et al., 2000; Van’t Boveneind-Vrubleuskaya et al., 2017). To prevent pollution, we regularly observe during the cultivation process. After the cultivation is completed, we also randomly sampled for 16S rDNA sequencing to identify the bacterial species.

### 2.4 Experimental grouping

We separated macrophages into two groups including the uninfected group and infected group, the former of which was free of MTB infection and the latter of which was infected with MTB. The number of intracellular MTB and ROS production were assessed at 0 h, 4 h, 24 h, and 48 h. RNA-seq employed macrophage samples at 24 h. At 48 h after MTB infection with macrophages, although ROS production had reached its peak, the number of macrophages surviving decreased. To ensure the quality and consistency of the sequencing samples, we chose a moderate 24 h. At least three different runs of each experiment were completed.

### 2.5 Measurement of Intracellular ROS

The macrophages were previously detached from the culture plates using trypsin (0.25%) and the cells were washed and resuspended in a serum-free medium. First, the cells were stained with the oxidative fluorescent dye probe from the reactive oxygen species assay kit (Beyotime, Shanghai, China) for 20 min at 37°C in 5% CO_2_ (Feng et al., 2017). Second, we determined the intracellular ROS using a flow cytometer (Guava^®^; Luminex, Austin, TX, USA) and analyzed the data using FlowJo™ software (Tree Star Inc., Ashland, OR, USA). The results were plotted as a histogram and analyzed, and the mean fluorescence intensities (MFIs) were compared.

### 2.6 Ribonucleic acid extraction

Total ribonucleic acid (RNA) was extracted from the cells using TRIzol^®^ Reagent (Invitrogen Life Technologies, Grand Island, NY, USA), according to the manufacturer’s instructions, and genomic DNA was removed using DNase I (TaKaRa, Beijing, China). RNA degradation and contamination were monitored on 1% agarose gels. RNA quality was then determined by using a 2100 Bioanalyser (Agilent Technologies, Inc., Santa Clara, CA, USA) and quantified using ND-2000 (NanoDrop™ Technologies). Only a high-quality RNA sample (OD_260/280 =_ 1.8–2.2, OD_260/230_ ≥ 2.0, RIN ≥ 8.0, 28S: 18S ≥ 1.0, > 1 μg) was used to construct the sequencing library.

### 2.7 Sequencing of libraries and quality control

Following the manufacturer’s instructions (Illumina, San Diego, CA), RNA purification, reverse transcription, and library creation were carried out at Shanghai Majorbio Bio-pharm Biotechnology Co., Ltd. (Shanghai, China). The Illumina NovaSeq 6000 sequencer (2 × 150-bp read length) was used to sequence the paired-end RNA-seq library. The raw paired-end reads were trimmed and quality controlled by fastp (https://github.com/OpenGene/fastp) (Chen et al., 2018) with default parameters. Then clean reads were separately aligned to the reference genome with the orientation mode using HISAT2 (http://ccb.jhu.edu/software/hisat2/index.shtml) (Kim et al., 2015) software. The mapped reads of each sample were assembled by StringTie (https://ccb.jhu.edu/software/stringtie/) in a reference-based approach (Pertea et al., 2015). Both the infected and uninfected groups had four samples. The number of clean reads per sample ranged from 47,707,378 to 62,104,790 (**Supplementary Table 1**).

### 2.8 Differential gene expression analysis and functional enrichment

The standard gene targets of ROS were analyzed and screened using three public databases (CTD, DISEASES, and GeneCards^®^). Through the human genome database GeneCards (https://www.genecards.org/) (Safran et al., 2010) we retrieved ROS-related human genes and created a gene set. We then analyzed the gene expression differences between macrophages infected and uninfected with MTB using the retrieved gene set by Venn. To identify DEGs (differential expression genes) between the two different samples/groups, the expression level of each gene was calculated according to the transcripts per million reads (TPM) method. RSEM (http://deweylab.biostat.wisc.edu/rsem/) (Li and Dewey, 2011) was used to quantify gene abundances. Essentially, differential gene expression analysis was performed using the DESeq2 (Love et al., 2014), and a *p*-adjust value ≤ 0.05 (DESeq2) was considered to be a significantly differentially expressed gene. In addition, functional enrichment analyses including GO (Gene Ontology; http://www.geneontology.org) and KEGG (Kyoto Encyclopedia of Genes and Genomes; http://www.genome.jp/kegg/) were performed to identify which DEGs were significantly enriched in GO terms and metabolic pathways at a *p*-adjust value ≤ 0.05 compared with the whole-transcriptome background. GO functional enrichment and KEGG pathway analyses were carried out by GOATOOLS (https://github.com/tanghaibao/Goatools) and KOBAS (http://kobas.cbi.pku.edu.cn/home.do) (Xie et al., 2011).

### 2.9 Protein network construction

String 11.5 (https://cn.string-db.org/) and Cytoscape v 3.9.1 software (USA) were used to analyze and draw a protein–protein interaction (PPI) map of the interactions between macrophages infected and uninfected with MTB and ROS-related gene expression proteins. Based on the String database information, the first 300 interaction groups with a combined score > 0.9 were selected and all excess interaction groups were filtered out (von Mering et al., 2005; Jacobs et al., 2023). To further identify the main differentially expressed proteins, we used the MCODE tool (the parameters were degree cutoff = 2; node sore cutoff = 0.2; k-core = 2; and max depth = 100) to screen out important subnetworks in Cytoscape.

### 2.10 Reverse transcription-quantitative polymerase chain reaction

According to GO, KEGG, and PPI analysis, we selected six genes related to ROS. The specific method is as follows. First, we identified genes related to oxidative function through GO analysis. Second, KEGG analysis was used to identify relevant pathways and identify genes related to ROS from upstream and downstream of the pathway. Third, and finally, we searched for specific possible relationships between genes and ROS in the GeneCards database (https://www.genecards.org/) under the condition of TPM (transcripts per million) > 1.

Total RNA was extracted at 24 h and was extracted from the cells using TRIzol^®^ Reagent (Invitrogen). Afterward, RNA was quantified using a micro ultraviolet spectrophotometer (Thermo; NanoDrop™ 2000), and the concentration was adjusted to be consistent. The RNA was reverse transcribed to cDNA using a Hifair™ II 1st Strand cDNA Synthesis SuperMix kit (Yeasen Biotech, China). The real-time quantitative PCR assays were performed using a Hifair qPCR SYBR™ Green Master Mix kit (Low ROX; Yeasen Biotech) on an ABI 7500 system (Applied Biosystems, USA). Primers were designed using Primer-BLAST (https://www.ncbi.nlm.nih.gov/tools/primer-blast) (**Table 1**). The expression of the genes was analyzed by the 2^−ΔΔCT^ method and was normalized to the expression of the β-actin (*ACTB*) gene. The difference between RT-qPCR and RNA-Seq data was compared using Pearson’s correlation coefficient tests (Luo et al., 2016).

**Table 1:** Primer sequences for differentially expressed gene validation.

Name	Forward	Reverse
*ACTB*	CATGTACGTTGCTATCCAGGC	CTCCTTAATGTCACGCACGAT
*CYBB*	ACCGGGTTTATGATATTCCACCT	GATTTCGACAGACTGGCAAGA
*ITPR1*	ATTGCTGGGGACCGTAATCC	TCCAATGTGACTCTCATGGCA
*CAMK2B*	GCACACCAGGCTACCTGTC	GGACGGGAAGTCATAGGCA
*GPX3*	GAGCTTGCACCATTCGGTCT	GGGTAGGAAGGATCTCTGAGTTC
*CAT*	TGTTGCTGGAGAATCGGGTTC	TCCCAGTTACCATCTTCTGTGTA
*SOD2*	TTTCAATAAGGAACGGGGACAC	GTGCTCCCACACATCAATCC

Name, gene symbol; Forward, forward primer for the gene (5′ to 3′ on the plus strand); Reverse, reverse primer for the gene (5′ to 3′ on the minus strand); ACTB, β-actin.

We used 10 mM *N*-acetylcysteine (NAC; Solarbio, Beijing, China) to treat the uninfected macrophages (the uninfected + NAC group) and infection macrophages (the infected + NAC group) (Wrotek et al., 2016). Then, we extracted total RNA at 24 h and performed RT-qPCR on six genes in the uninfected group, infected group, uninfected + NAC group, and infected + NAC group, as mentioned above. Finally, we calculated and plotted a histogram.

### 2.11 Statistical analysis

The data shown in graphs were presented as means ± SEMs. The number of experimental replicates is indicated in the figure legend. The statistical analyses were performed with GraphPad Prism 9.0 software (GraphPad Software Inc., La Jolla, CA, USA) using either unpaired two-tailed *t*-tests for comparison between the two groups or one-way ANOVA. The Pearson correlation coefficient tests were analyzed by the software package of SPSS version 24.0 software (IBM Corporation, Armonk, NY, USA). The statistical differences were considered significant when the *p*-value was *<* 0.05, with asterisks denoting the degree of significance (*, *p <* 0.05; **; *p <* 0.01; ***, *p <* 0.001; and ****, *p <* 0.0001).

## 3 Results

### 3.1 Intracellular bacteria and ROS in MTB-infected macrophages

**Figure 1:**
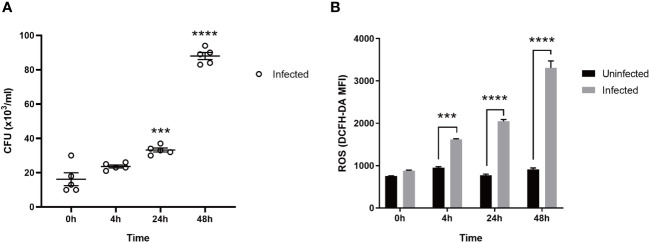
The CFU and ROS in MTB-infected macrophages. **(A)**: CFU. **(B)**: ROS. Uninfected group, macrophages not infected with MTB; infected group, macrophages infected with MTB; ROS, reactive oxygen species; CFU, colony-forming units. MTB, *Mycobacterium tuberculosis*; MFI, mean fluorescence intensity; DCFH-DA, 2,7-dichlorodihydrofluorescein diacetate. The values are presented as the mean ± SEM. ***, *p <* 0.001; ****, *p <* 0.0001. The asterisk (*) in **A** is compared with 0 h. At least three different runs of each experiment were completed.

As shown in **Figure 1A**, the bacteria in macrophages infected with MTB gradually increased in number with the prolongation of the post-infection time. As shown in **Figure 1B**, the ROS production of the uninfected group did not change significantly over 0 h, 4 h, 24 h, and 48 h. However, after the macrophages were infected with MTB, ROS levels increased at 4 h and peaked at 48 h, higher than the uninfected group (*p <* 0.05).

### 3.2 Differential expression of genes related to ROS in MTB-infected macrophages

**Figure 2:**
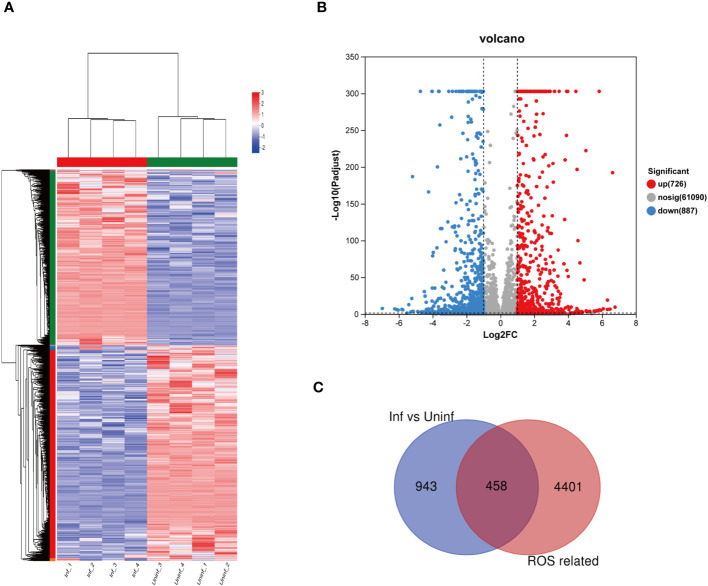
Differential expression of the genes related to ROS in MTB-infected macrophages. **(A)** a clustering heat map of the differentially expressed genes. **(B)** a volcano map of differential gene expression, The horizontal axis represents the fold change value (FC); the vertical axis represents the *p*-adjust value. The values of the horizontal and vertical coordinates have been logarithmized. The dot represents a specific gene; the red dots represent the upregulated genes; the blue dots represent the downregulated genes; and the gray dots represent the non-significantly differentially expressed genes. *p*-adjust value < 0.05 is the difference threshold; FC ≥ 2 and FC ≤ 0.5 was used as a cutoff value to color genes. **(C)** differentially expressed genes and ROS-related genes, as identified via Venn analysis. Uninfected group, macrophages not infected with MTB; infected group, macrophages infected with MTB; inf, infected group; uninf, uninfection group; ROS, reactive oxygen species.

As shown in **Figures 2A, B**, RNA-seq obtained 1,613 differentially expressed genes in macrophages infected and uninfected with MTB, including 726 upregulated genes and 887 downregulated genes. Among the 1,613 differentially expressed genes, 458 differentially expressed genes related to ROS were revealed between the macrophages infected and uninfected with MTB through Venn analysis, as shown in **Figure 2C**.

We conducted GO analysis and KEGG analysis on 1,613 genes, except for the 458 ROS-related genes. As shown in **Supplementary Figure 1A**, the results of the GO annotation analysis indicated the influence of genes on three aspects. As for molecular functions, they primarily affected binding, catalytic activity, and molecular function regulators. As for cellular components, they primarily affected the function of organelles, followed by the function of cell membranes. As for biological processes, they primarily affected cellular processes, biological regulation, and metabolic processes. As shown in **Supplementary Figure 1B**, that is, the results of the GO enrichment analysis, the differentially expressed genes are mainly related to cell adhesion, cell motility, cell junctions, and calcium ion binding. As shown in **Supplementary Figure 2**, the KEGG annotation analysis results revealed that the genes were primarily related to infectious diseases, and cancer in terms of human diseases. They affected the immune system and circulatory system in terms of organismal systems. They affected eukaryotes and cell motility in terms of cell processes. They affected cytokine–cytokine receptor interactions, the PI3K-Akt signaling pathway, and the Wnt signaling pathway in terms of environmental information processing. The KEGG enrichment analysis did not enrich into pathways with a *p*-adjust value < 0.05.

### 3.3 GO analysis of genes related to ROS in MTB-infected macrophages

**Figure 3:**
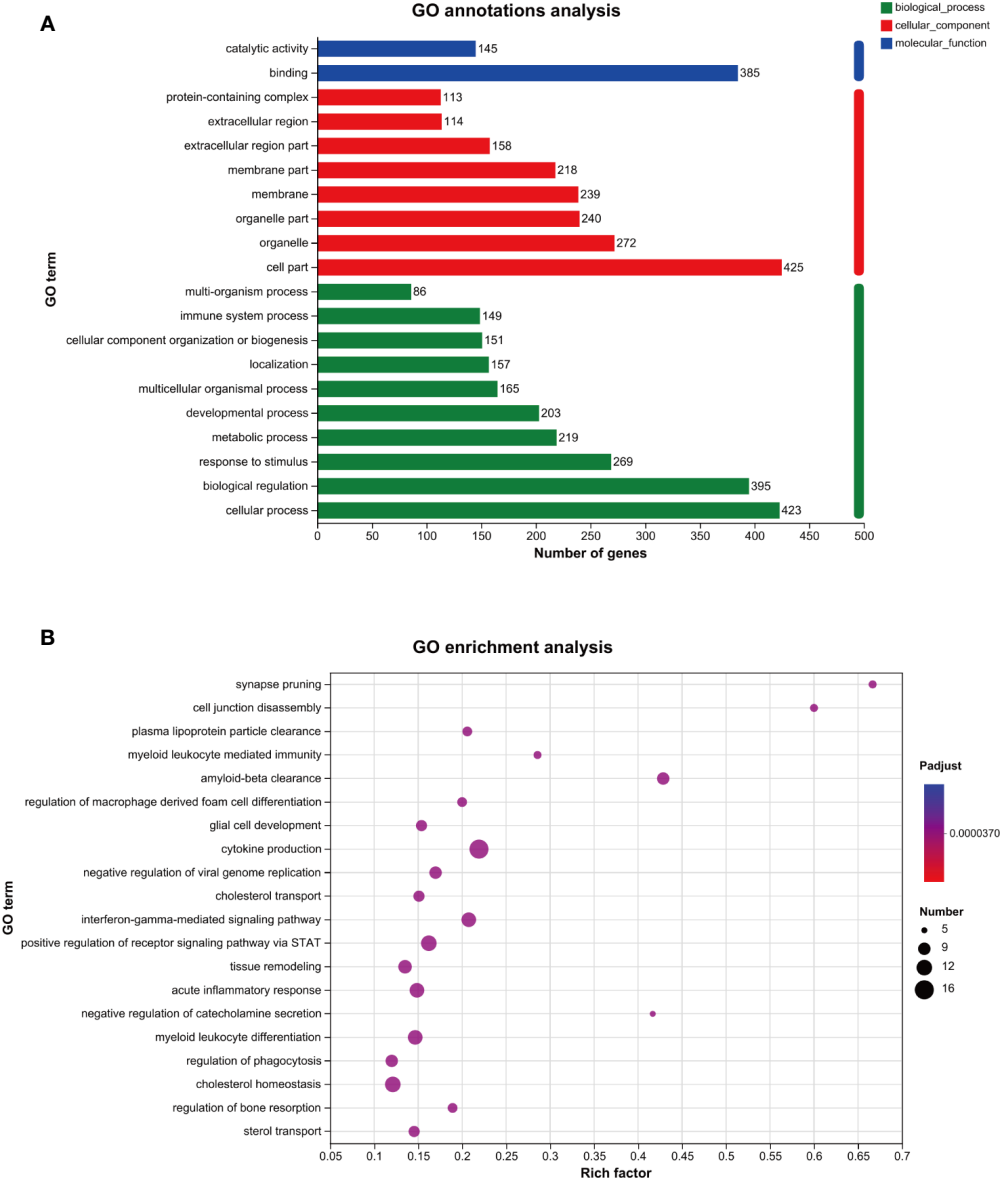
GO analysis of the genes related to ROS in MTB-infected macrophages. **(A)**: GO annotations analysis. **(B)**: GO enrichment analysis. The horizontal axis represents the rich factor (i.e., the number of genes in the GO term/total number of genes); the vertical axis represents the GO term; the size of the dot represents the number of genes in this GO term; and the color of the dot represents the *p-*adjust value. Only the GO enrichment results for the top 20 with a *p*-adjust value < 0.05 are shown. GO, Gene Ontology; MTB, *Mycobacterium tuberculosis*; ROS, reactive oxygen species.

As shown in **Figure 3A**, the results of the GO annotation analysis indicated the influence of the 458 differentially expressed genes on three aspects. As for molecular functions, they primarily affected binding and catalytic activity. As for cellular components, they primarily affected the function of organelles, followed by the function of cell membranes. As for biological processes, they primarily affected cellular processes, biological regulation, and responses to stimuli. As shown in **Table 2**, among the 458 differentially expressed genes, 269 were related to receiving external stimuli. From the perspective of cell composition, 272 differentially expressed genes were associated with organelles, which confirms that mitochondria, endoplasmic reticulum, and other related organelles were related to the production of ROS in macrophages infected with MTB. As for molecular function, some differentially expressed genes expressed some enzymes related to ROS, such as *PTGS2, GPX3, SOD2, CAT, ALDH1A2*, and *CHAC1*.

**Table 2:** Partial GO annotation analysis.

Term description	Term type	Number	Gene names
Cellular process	Biological process	423	See **Supplementary Table 2**
Biological regulation	Biological process	395	See **Supplementary Table 2**
Response to stimulus	Biological process	269	See **Supplementary Table 2**
Immune system process	Biological process	149	See **Supplementary Table 2**
Organelle	Cellular component	272	See **Supplementary Table 2**
Membrane	Cellular component	240	See **Supplementary Table 2**
Binding	Molecular function	385	See **Supplementary Table 2**
Catalytic activity	Molecular function	145	See **Supplementary Table 2**
Antioxidant activity	Molecular function	8	See **Supplementary Table 2**

GO, Gene Ontology; Term description, GO secondary classification term; Term type, GO primary classification name; Number, the number of genes annotated to the secondary classification function of the GO; Gene names, the name of the genes annotated to the secondary classification function of the GO.

The GO enrichment analysis revealed that the genes above the primary functions were enriched in cytokine production, acute inflammatory response, and cholesterol homeostasis (**Figure 3B**).

### 3.4 KEGG analysis of genes related to ROS in MTB-infected macrophages

**Figure 4:**
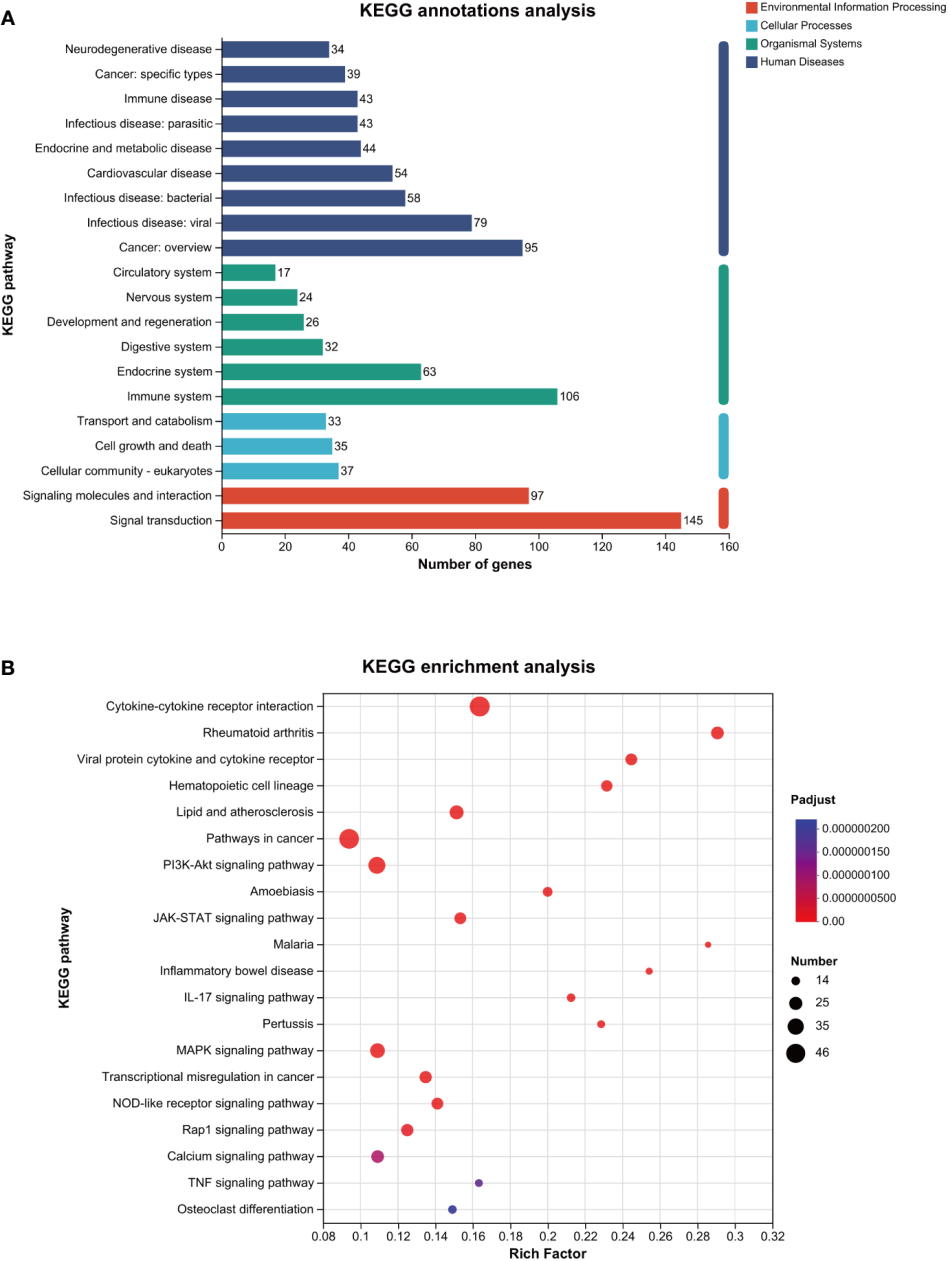
KEGG analysis of the genes related to ROS in MTB-infected macrophages. **(A)**: KEGG annotations analysis. **(B)**: KEGG enrichment analysis. The horizontal axis represents the rich factor (i.e., the number of genes enriched in the KEGG path/total number of genes); the vertical axis represents the KEGG pathway; the size of the dot represents the number of genes in this KEGG path; and the color of the dot represents the *p-*adjust value. Only the KEGG enrichment results for the top 20 with a *p*-adjust value < 0.05 are shown. KEGG, Kyoto Encyclopedia of Genes and Genomes; MTB, *Mycobacterium tuberculosis*; ROS, reactive oxygen species.

As shown in **Figure 4A**, the KEGG annotation analysis results revealed that the aforementioned differentially expressed genes were primarily related to the pathogenesis of infectious diseases, cancer, and cardiovascular diseases in terms of human diseases, affected the immune system, endocrine system, and digestive system in terms of biological systems, and affected eukaryotic cells, cell growth, and death in terms of cell processes.

As shown in **Figure 4B** and **Table 3**, the KEGG enrichment analysis revealed that it primarily affected the IL-17 signaling pathway and the NOD-like receptor pathway in the biological system, rheumatoid arthritis, atherosclerosis, and cancer in disease, and affected cytokine and receptor interactions and the PI3K-AKT, JAK-STAT, MAPK, and Rap1 pathways in the signal pathway. In addition, there was a related pathway, FoxO, in which the differentially expressed genes might involve upstream *SGK* and downstream *CAT*, *MnSOD*, and other genes closely related to antioxidant effects.

**Table 3:** Partial KEGG enrichment analysis.

Pathway description	Number	Gene names
PI3K-Akt signaling pathway	36	See **Supplementary Table 3**
MAPK signaling pathway	30	See **Supplementary Table 3**
Lipid and atherosclerosis	28	See **Supplementary Table 3**
Calcium signaling pathway	25	See **Supplementary Table 3**
Rap1 signaling pathway	24	See **Supplementary Table 3**
JAK-STAT signaling pathway	23	See **Supplementary Table 3**
TNF signaling pathway	16	See **Supplementary Table 3**
FoxO signaling pathway	15	See **Supplementary Table 3**

KEGG, Kyoto Encyclopedia of Genes and Genomes; Pathway description, a specific description of the KEGG pathway; Number, the number of genes or transcripts enriched into this pathway; Gene names, the name of the gene enriched in this pathway.

### 3.5 PPI analysis of genes related to ROS in MTB-infected macrophages

**Figure 5:**
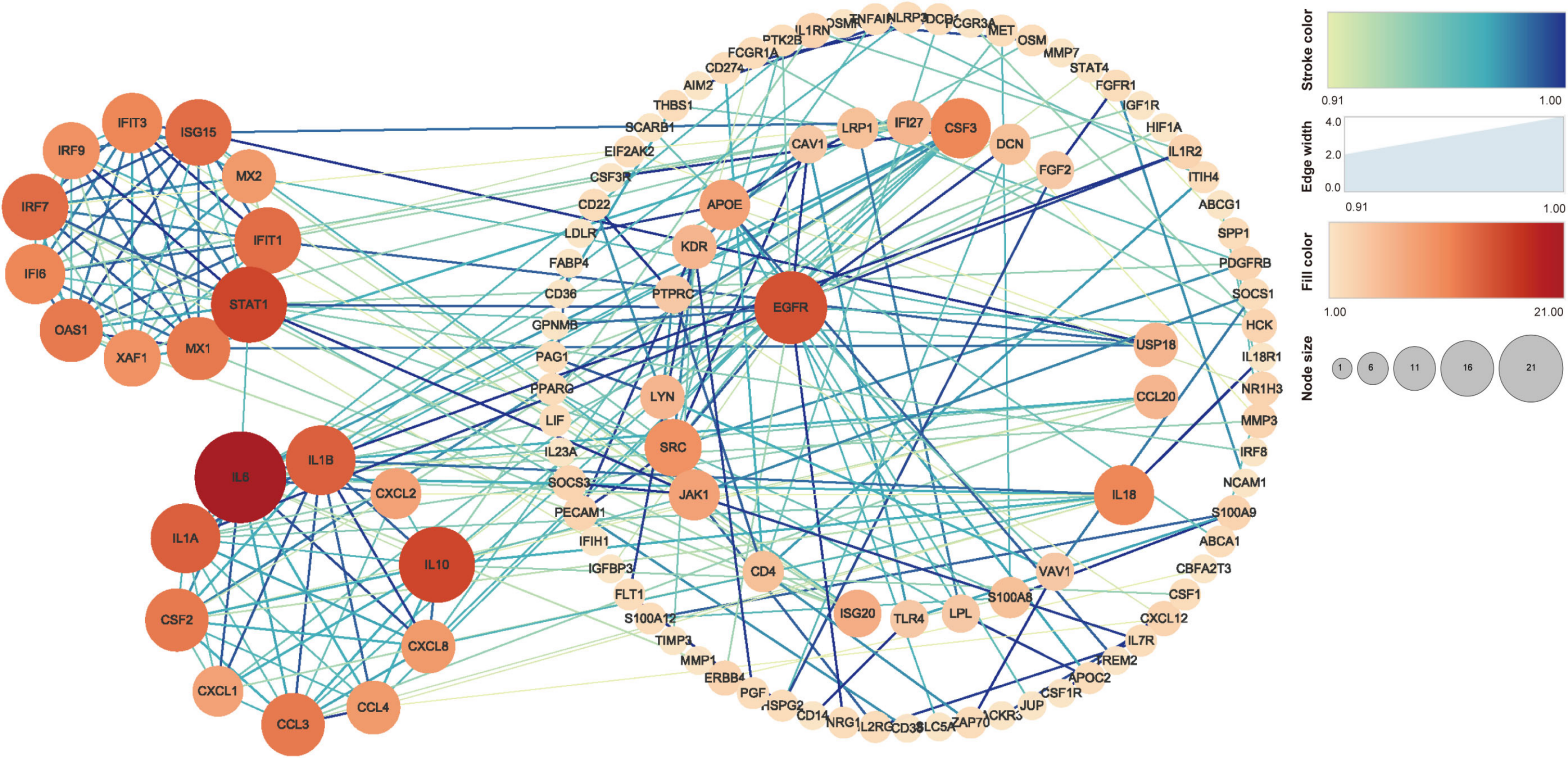
PPI analysis of the genes related to ROS in MTB-infected macrophages. The stroke color and edge width represent the combined score (confidence calculated using several types of evidence). The fill color and node size represent the number of related proteins. PPI, protein–protein interaction; MTB, *Mycobacterium tuberculosis*; ROS, reactive oxygen species.

The PPI analysis in **Figure 5** shows that IL-6 has 21 related proteins and has the most protein interactions. Subsequent sub-analysis yielded two sub-rings, one of which contained proteins such as IL-6, IL-1A, IL-1B, CXCL2, IL-10, CXCL8, CCL4, CCL3, CXCL1, and CSF2, and the other of which contained proteins such as STAT1, ASG15, IRF7, OAS1, IFIT1, IFIT3, IRF9, IFI6, XAF1, and MX1.

### 3.6 Validation of differentially expressed genes related to ROS in macrophages

**Figure 6:**
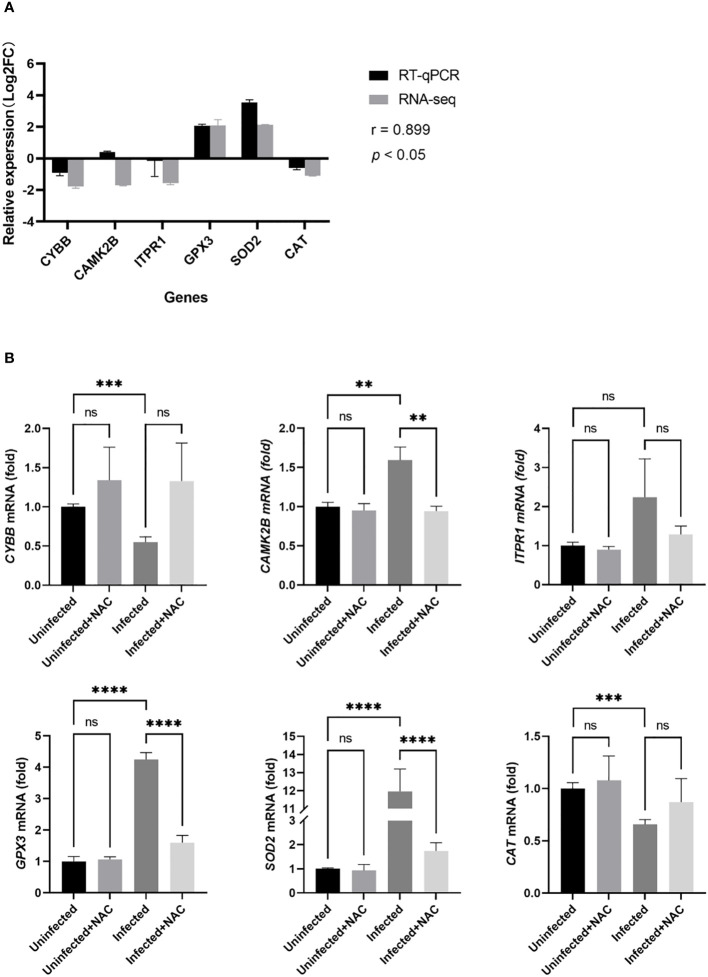
RT-qPCR validation of the ROS-related genes in MTB-infected macrophages. **(A)**: validation of the RNA-seq data by RT-qPCR analysis. The *x*-axis represents the gene name; the *y*-axis represents the relative expression level expressed as log2 (fold change) in gene expression. The relative expression of the six genes was determined by RT-qPCR and compared with the results of RNA-seq. We calculated the Pearson correlation coefficient (*r*) between the different methods for all transcripts. The correlation coefficient was 0.899 with a *p*-value < 0.05. **(B)**: RT-qPCR was used to measure the gene expression of macrophages in the uninfected group, the infected group, the uninfected + NAC group, and the infected + NAC group. Uninfected group, macrophages not infected with MTB; infected group, macrophages infected with MTB; uninfected + NAC group, macrophages not infected with MTB but treated with NAC; infected + NAC group, macrophages infected with MTB and treated with NAC. NAC, *N*-acetylcysteine; RNA-seq, RNA sequencing; RT-qPCR, reverse transcription-quantitative polymerase chain reaction. The values are presented as the mean ± SEM (*n* = 3). **, *p <* 0.01; ***, *p <* 0.001; ****, *p <* 0.0001.

To verify the accuracy of the RNA-seq data, six genes were chosen for RT-qPCR. As shown in **Figure 6A**, the expression of most of the genes was consistent with RNA-seq based on the Pearson correlation coefficient value (*r* = 0.899, *p* < 0.05). The difference between RT-qPCR and RNA-Seq data might be explained by a different sensitivity to gene expression in these two methods (Zhao et al., 2014). As is shown in **Figure 6B**, for the genes related to ROS production, the expression levels of *CAMK2B* in the infected groups were significantly higher than those in the uninfected group (*p <* 0.05), whereas the expression levels of *CYBB* were significantly lower than that in the uninfected group (*p <* 0.05). The levels of *ITPR1* in the infected groups were close to those in the uninfected group (*p* > 0.05). For the genes related to ROS clearance, the expression levels of *GPX3* and *SOD2* in the infected groups were significantly higher than those in the uninfected group (*p <* 0.05), whereas the expression levels of *CAT* were significantly lower than that in the uninfected group (*p <* 0.05). There was no significant difference in the expression of six genes between the uninfected group and the uninfected + NAC group (*p >* 0.05). The expression of *CAMK2B, GPX3*, and *SOD2* in the infection + NAC group was lower than that in the infection group (*p <* 0.05).

## 4 Discussion

The above results show that after MTB infection, with the prolongation of time, the number of intracellular bacteria in macrophages increased and ROS increased (**Figure 1**). This suggests that MTB infection is one of the reasons why macrophages produce OS. However, there is limited research on the genes and transcriptomics involved in MTB-induced OS. In this study, we identified 1,613 differentially expressed genes related to MTB infection through RNA-seq and transcriptomics analysis (**Figures 2A, B**). A Venn analysis was conducted on the ROS-related genes in the GeneCards database and 1,613 differentially expressed genes. Subsequently, 458 differentially expressed genes were obtained (**Figure 2C**), and GO, KEGG, and PPI analyses were performed.

According to the GO annotation analysis (**Figure 3A**), more than 50% of the 458 ROS-related differentially expressed genes obtained in this study act on the cell membrane and organelles (**Table 2**). This is consistent with the research findings that organelles, such as mitochondria and endoplasmic reticulum, can produce ROS (Zorov et al., 2014). However, there is limited research on ROS production in macrophage organelles infected with MTB. The results of the GO analysis may indicate that MTB, similar to other pathogens, can produce ROS through organelles (Rhen, 2019; Wang et al., 2022). In terms of biological processes, over 50% of the genes are involved in cellular processes, biological regulation, and responses to stimuli. It is suggested that MTB infection can stimulate the occurrence of OS in macrophages, leading to an increase in intracellular ROS. The ROS may lead to changes in macrophage function and survival. As stated in the study, ROS can lead to autophagy and apoptosis through multiple pathways (Kaminskyy and Zhivotovsky, 2014). In terms of molecular function, 84% of the differentially expressed genes involve binding and 32% involve catalytic activity. There are also eight genes involved in antioxidant activity, most of which are regulatory genes for antioxidant enzymes. It is suggested that MTB infection can stimulate an increase in the level of antioxidant enzyme regulatory genes to adapt to the increase in OS levels, eliminate ROS, and promote redox balance. The above-mentioned genes’ primary activities were enriched in cytokine generation, acute inflammatory response, and cholesterol homeostasis according to GO enrichment analysis (**Figure 3B**). MTB infection can cause inflammatory reactions, and when ROS is present, the lipid components on the cell membrane may suffer oxidative damage. Therefore, exploring and regulating ROS is particularly important for balancing host damage and sterilization.

The KEGG annotation analysis showed that 458 ROS-related differentially expressed genes were mainly involved in signal transmission, infectious diseases, cell growth and death, and the immune system (**Figure 4A**). The analysis suggests that MTB infection can affect the survival of host cells through immune responses and related signaling pathways. The KEGG enrichment analysis shows that ROS-related differentially expressed genes are enriched in the IL-17 signaling pathway and NOD-like receptor signaling pathway of the organ system, both of which are related to ROS (**Figure 4B**). Some studies have shown that ROS promotes the production of IL-17 (He et al., 2016), but others suggest that IL-17 can induce ROS production and cause cell apoptosis (Zhou et al., 2018). Nucleotide-binding oligomerization domain-like receptors (NOD-like receptors, NLRs) contain many members, among which nod-like receptor protein 1 (NLRP1) and NLRP3 are more representative. ROS may induce the NLRP3 (Kelley et al., 2019), thereby regulating cell death (Huang et al., 2021). ROS-related differentially expressed genes are enriched in the PI3K-AKT, JAK-STAT, and MAPK signaling pathways, involving 36, 23, and 30 related genes, respectively (**Table 3**). This suggests that the increase in ROS in macrophages caused by MTB infection may be related to these signaling pathways. Among them, the JAK-STAT pathway is one of the most significant differences; it is also associated with cell apoptosis. Additionally, a related pathway called FoxO has differentially expressed genes that may include downstream *CAT*, upstream *SGK*, *MnSOD*, and other genes that are closely related. As a result, ROS-related differential y expressed genes may be crucial for controlling immunological function and macrophage cell apoptosis.

We conducted PPI enrichment analysis on proteins expressed by 458 genes to explore important proteins and their interactions, including biological signal transduction, gene expression regulation, energy metabolism, and cell cycle regulation. We conducted an enrichment analysis on the proteins expressed by the 458 genes (**Figure 5**). Two major subgene clusters were produced. The protein expressed in the first gene cluster corresponds to cytokine responses after infection, while that in the second gene cluster is closely related to interferon responses and transmission and defense against viruses and other immune responses. Among them, IL-6 has a significant interaction relationship. As is well known, IL-6 expressed by *IL-6* is an effective multifunctional cytokine encoded by the human interleukin 6 gene. IL-6 can regulate liver OS and it can participate in the JAK/STAT signaling pathway through IL-6 receptors (Hassan et al., 2014). Another interaction is STAT1, which is related to the JAK-STAT pathway screened in the KEGG analysis, which may further indicate a close relationship between OS caused by MTB infection of macrophages and host apoptosis.

We also conducted GO and KEGG analyses on 1,613 genes that removed 458 genes. The functions of these genes may be related to cell migration. The bactericidal process of macrophages is closely related to cell migration. In the KEGG annotation analysis, these genes may be involved in the PI3K-Akt and Wnt signaling pathways. However, during the KEGG enrichment analysis, the *p*-adjust value of < 0.05 was not enriched in the signaling pathway. This may be related to excluding ROS-related genes. ROS not only causes oxidative stress but also participates in many signaling pathways (Dhanasekaran and Reddy, 2008). The mechanism of killing MTB may be directly or indirectly related to ROS. OS seemed to be driving most of the transcriptional changes involved in macrophages infected by MTB.

**Figure 7:**
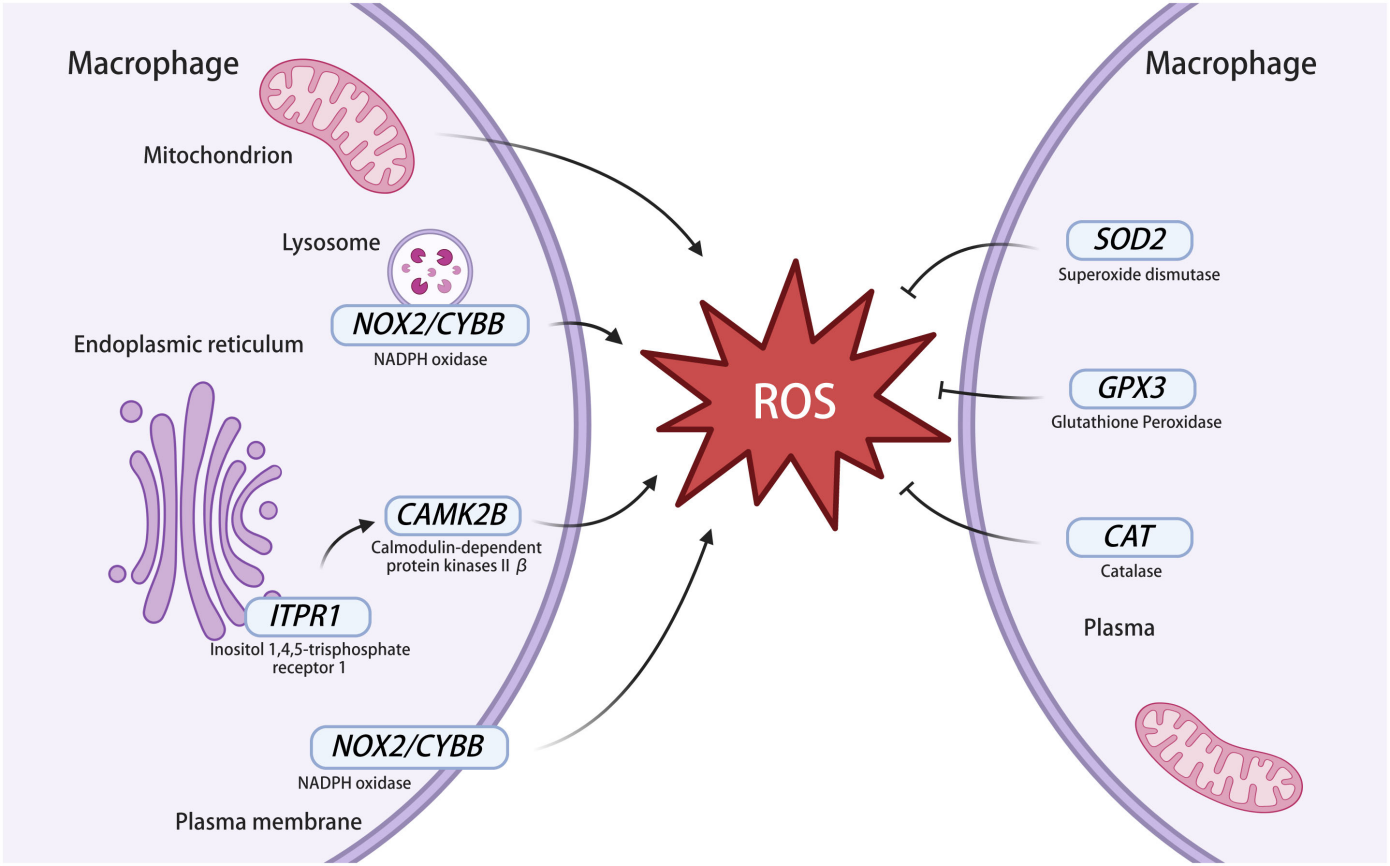
ROS-related genes in macrophages infected with MTB. The left part of the figure represents genes involved in ROS production and the right part of the figure represents genes involved in ROS clearance*. NOX2* exists in the membrane of macrophages and lysosomes and can express NADPH oxidase to produce ROS*. NOX2* is also known as *CYBB*. *ITPR1* is an intracellular reticulum calcium channel activated by inositol triphosphate, which leads to ROS generation through a CAMKII. CAMKII contains multiple subunits, including CAMKIIβ and it is expressed by *CAMK2B*. The superoxide dismutase expressed by *SOD2* acts on ROS to generate H_2_O_2_, then H_2_O_2_ is decomposed by catalase, which is expressed by *CAT*, and glutathione peroxidase is expressed by *GPX3* to clear ROS. NADPH oxidase, nicotinamide adenine dinucleotide phosphate oxidase; ROS, reactive oxygen species; CAMKII, calmodulin-dependent kinase II. This image was created using BioRender.com.

The research results (**Figure 6**) show that, among the genes related to ROS production, *CAMK2B* infected with MTB are highly expressed, *CYBB* is expressed at low levels, and the expression of *ITPR1* is similar to that of uninfected macrophages. This indicates that MTB infection can affect the expression of ROS-related genes and regulate OS in macrophages. However, when we used NAC to treat macrophages infected and uninfected with MTB, only *CAMK2B, GPX3*, and *SOD2* showed significant differences. The high expression levels of these three genes may lead to significant differences. It is also possible that the proteins expressed by these three genes were more closely related to the production, clearance, or other functions of ROS. All the genes also require further experimental validation to ensure their association with OS. Among them, *CYBB* is the main component of the phagocytic microbicidal oxidase system. *CYBB* is also known as *NOX2*. Gp91^phox^ encoded by *CYBB* is a key functional subunit of the nicotinamide adenine dinucleotide phosphate oxidase (NADPH oxidase) complex. *NOX2* on the lysosomal and plasma membranes of cells contributes to killing of microbes (Grauers Wiktorin et al., 2020) (**Figure 7**). The ROS derived from *NOX2* has been shown to have immunomodulatory effects (Sareila et al., 2011). The downregulation of *CYBB* expression after MTB infection may be due to a decrease in ROS production caused by the body’s self-protection mechanisms after a large amount of ROS is produced. Calmodulin-dependent kinase II (CAMKII) is an important multifunctional serine/threonine protein kinase. There are four different homologous types of CaMKII (α, β, γ, and δ), which are encoded by four independent genes *CaMK2*(*A, B, G*, and *D*). There are few studies on tuberculosis related to CAMKII, but studies on other diseases show that CAMKII is closely related to ROS production (Sanders et al., 2013). CAMKII may also become an important target for regulating ROS. *ITPR1* is an intracellular reticulum calcium channel activated by inositol triphosphate, which leads to ROS generation through a CAMKII (**Figure 7**). In the validation experiment, there was no significant difference in the expression of *ITPR1* before and after MTB infection, which may be due to the low expression level of the gene. The results also showed the expression of genes related to ROS clearance. Human antioxidant enzymes mainly include superoxide dismutase (Wang et al., 2018), catalase, and glutathione peroxidase (Singel and Segal, 2016) (**Figure 7**). The genes *GPX3, SOD2*, and *CAT* of the above three enzymes were verified by RT-qPCR, and it was found that the expression of *GPX3* and *SOD2* increased after infection, whereas the expression of *CAT* decreased. Many studies (Ren et al., 2021; Wan et al., 2023) have shown that *SOD2* is upregulated and regulates ROS in macrophages infected with MTB, but there are few reports of *GPX3* and *CAT*. Among them, the high expression of *GPX3* and *SOD2* may be an adaptive change of macrophages in response to OS caused by MTB, to reduce ROS levels, maintain the normal function of macrophages, and prevent death. In addition, the reverse changes in *CAT*, *SOD2*, and *GPX3* after 24 h of MTB infection may also be related to our failure to observe the levels of these three genes at other time points. Perhaps after MTB stimulation, the expression levels of these three genes are time dependent and the trend of change is not consistent, and the reasons for this need to be explored.

Based on verification, *CAMK2B*, *GPX3*, and *SOD2* may be associated with ROS. They may become new targets for the treatment of tuberculosis. First, ROS may directly or indirectly affect the survival and death of macrophages, thereby affecting the killing of MTB (Kaminskyy and Zhivotovsky, 2014). We explore the antagonists or agonists of these differentially expressed genes that may contribute to the killing of MTB in the host, thereby improving the cure rate of tuberculosis. Second, research shows that ROS could cause tissue damage (Shastri et al., 2018). By regulating the above genes, the level of ROS could be reduced, thereby helping to reduce tissue damage. In addition, antituberculosis drugs have serious side effects and economic burdens in the treatment of drug-resistant tuberculosis (Pant et al., 2023). We could also combine regulatory genes with antituberculosis drugs. Antioxidant therapy may assist in antituberculosis treatment, thereby improving efficacy.

In this study, although we found that MTB infection of macrophages can produce many ROS-related differentially expressed genes, we only selected six differentially expressed genes for RT-qPCR validation at 24 h. We did not validate at the peak of infection and protein levels, nor did we explore the relevant molecular mechanisms. In future research, we will refine the relevant research content mentioned above and conduct *in vivo* experiments to obtain more detailed data.

## 5 Conclusion

In conclusion, the ROS-related differentially expressed genes between MTB infected and uninfected macrophages may be related to some organelles and involved in various biological processes, molecular functions, and signaling pathways. Among them, *CAMK2B*, *GPX3*, and *SOD2* may be related to ROS. The discovery of these genes will contribute to the study of potential new targets for host-targeted therapy of tuberculosis.

## Data availability statement

The datasets presented in this study can be found in online repositories. The names of the repository/repositories and accession number(s) can be found below: https://www.ncbi.nlm.nih.gov/, PRJNA960665.

## Ethics statement

Ethical approval was not required for the studies on humans in accordance with the local legislation and institutional requirements because only commercially available established cell lines were used.

## Author contributions

RS: Data curation, Formal analysis, Investigation, Methodology, Software, Validation, Visualization, Writing – original draft. JY: Writing – review & editing, Methodology, Validation, Formal analysis, Software. TG: Investigation, Methodology, Formal analysis, Software, Writing – review & editing. YL: Formal analysis, Investigation, Writing – review & editing, Methodology, Software. WS: Writing – review & editing, Investigation, Formal analysis, Methodology, Software. YW: Investigation, Resources, Writing – review & editing, Formal analysis, Software. YP: Conceptualization, Methodology, Project administration, Supervision, Writing – review & editing, Funding acquisition, Resources. QL: Conceptualization, Data curation, Funding acquisition, Project administration, Resources, Supervision, Writing – review & editing.
