# Supplementary material for: Selection and validation of genes related to oxidative stress production and clearance in macrophages infected with Mycobacterium tuberculosis
Source: Front Cell Infect Microbiol. 2023 Dec 12;13:1324611. doi: 10.3389/fcimb.2023.1324611 (PMC10749926; doi:10.3389/fcimb.2023.1324611)
Supplement: Supplementary file 1 [file Image_1.pdf]

Supplementary Material

Supplementary Figures

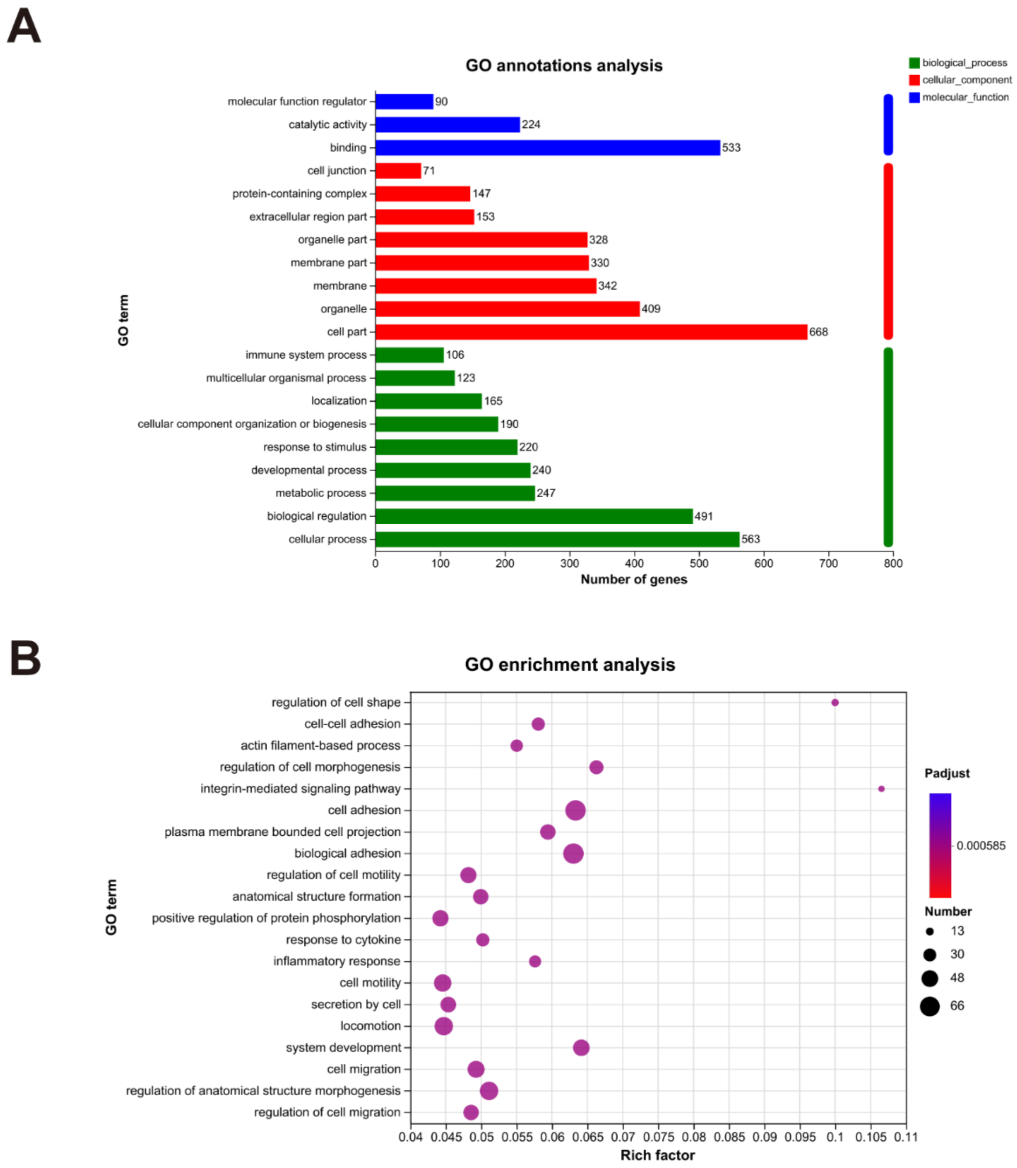

Supplementary Figure 1

GO analysis of differential genes in macrophages infected and uninfected with MTB except for 458 ROS-related genes. (**A**): GO annotations analysis. (**B**): GO enrichment analysis. The horizontal axis represents the rich factor (i.e., the number of genes in GO term/total number of genes); the vertical axis represents the GO term; the size of the dot represents the number of genes in this GO term; the color of the dot represents the *p*-adjust value. Only the GO enrichment results for the top 20 under a *p*-adjust value < 0.05 are shown. GO, Gene Ontology; MTB, *Mycobacterium tuberculosis*; ROS, reactive oxygen species.

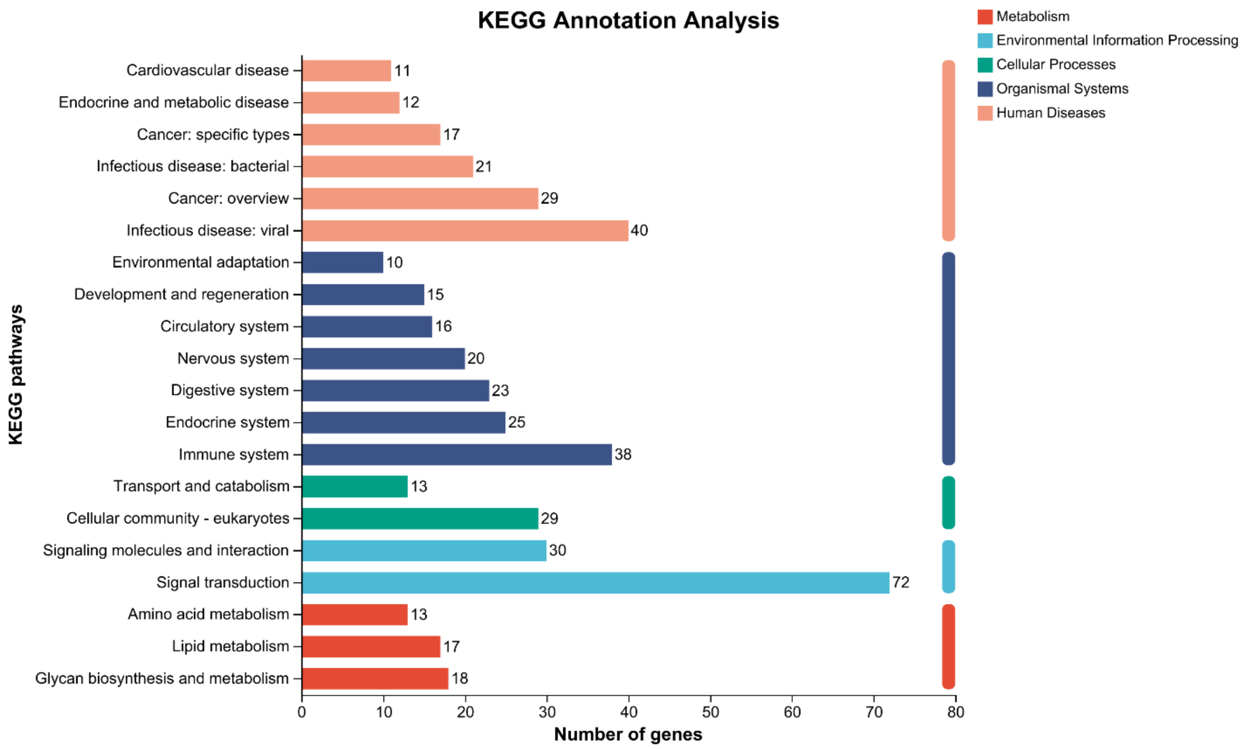

Supplementary Figure 2

KEGG annotations analysis of differential genes in macrophages infected and uninfected with MTB except for 458 ROS-related genes. KEGG, Kyoto Encyclopedia of Genes and Genomes; MTB, *Mycobacterium tuberculosis*; ROS, reactive oxygen species.
